# Investigating effects of sodium beta‐hydroxybutyrate on metabolism in placebo‐controlled, bilaterally infused human leg with focus on skeletal muscle protein dynamics

**DOI:** 10.14814/phy2.15399

**Published:** 2022-08-19

**Authors:** Henrik Holm Thomsen, Jonas Franck Olesen, Rasmus Aagaard, Bent Roni Ranghøj Nielsen, Thomas Schmidt Voss, Mads Vandsted Svart, Mogens Johannsen, Niels Jessen, Jens Otto L. Jørgensen, Nikolaj Rittig, Ermina Bach, Niels Møller

**Affiliations:** ^1^ Department of Internal Medicine, Clinic for Diabetes and Endocrinology Viborg Regional Hospital Viborg Denmark; ^2^ Department of Internal Medicine and Endocrinology Aarhus University Hospital Aarhus Denmark; ^3^ Research Unit for Multimorbidity Viborg Regional Hospital Viborg Denmark; ^4^ Department of Anesthesiology Randers Regional Hospital Randers Denmark; ^5^ Department of Cardiology Aarhus University Hospital Aarhus Denmark; ^6^ Steno Diabetes Center Aarhus Aarhus University Hospital Aarhus Denmark; ^7^ Department of Forensic Medicine, Bioanalytical Unit Aarhus University Aarhus Denmark; ^8^ Department of Biomedicine Aarhus University Aarhus Denmark

**Keywords:** amino acid tracer, beta‐hydroxybutyrate, ketone bodies, ketosis, perfused leg

## Abstract

Systemic administration of beta‐hydroxybutyrate (BHB) decreases whole‐body protein oxidation and muscle protein breakdown in humans. We aimed to determine any direct effect of BHB on skeletal muscle protein turnover when administered locally in the femoral artery. Paired design with each subject being investigated on one single occasion with one leg being infused with BHB and the opposing leg acting as a control. We studied 10 healthy male volunteers once with bilateral femoral vein and artery catheters. One artery was perfused with saline (Placebo) and one with sodium‐BHB. Labelled phenylalanine and palmitate were used to assess local leg fluxes. Femoral vein concentrations of BHB were significantly higher in the intervention leg (3.4 (3.2, 3.6) mM) compared with the placebo‐controlled leg (1.9 (1.8, 2.1) mM) with a peak difference of 1.4 (1.1, 1.7) mM, *p* < 0.0005. Net loss of phenylalanine for BHB vs Placebo −6.7(−10.8, −2.7) nmol/min vs −8.7(−13.8, −3.7) nmol/min, *p* = 0.52. Palmitate flux and arterio‐venous difference of glucose did not differ between legs. Under these experimental conditions, we failed to observe the direct effects of BHB on skeletal muscle protein turnover. This may relate to a combination of high concentrations of BHB (close to 2 mM) imposed systemically by spillover leading to high BHB concentrations in the saline‐infused leg and a lack of major differences in concentration gradients between the two sides—implying that observations were made on the upper part of the dose–response curve for BHB and the relatively small number of subjects studied.

## Graphical abstract

Investigation of direct effects on skeletal muscle protein turnover of beta‐hydroxybutyrate infused in one leg when opposing leg acts as control. Due to spill‐over and low number of participants no significant effects were observed. Caution to spillover effects are warranted in futures studies.

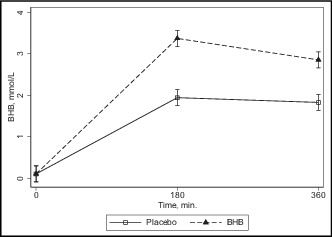

## 1 INTRODUCTION

Loss of skeletal muscle has numerous detrimental effects on overall health and prognosis (Anker et al., 1997; Kalantar‐Zadeh et al., 2013). Skeletal muscle loss can be a consequence of both acute and chronic illness due to inflammation, immobilization, insufficient energy, and protein intake. Hitherto, the focus has predominantly been on protein, lipid, and carbohydrate supplementation, but recently, studies on exogenous beta‐hydroxybutyrate (BHB), a lipid intermediate, has attracted interest with proposed protein‐sparing capabilities (Møller, 2020). Endogenous ketone bodies are primarily produced in the liver when carbohydrate availability is sparse, for example, prolonged fasting, ketogenic diets, exercise, or diabetic ketoacidosis. The resultant rise in acetoacetate and BHB stems from the abundant beta‐oxidation of nonesterified fatty acids (NEFA) and several organs are effectively fueled by BHB during ketotic states (Gormsen et al., 2017; Svart et al., 2018). With the provision of exogenous ketone salts or BHB precursor compounds, a state of ketosis can be obtained without the inconveniences of prolonged fasting and/or ketogenic diets (Stubbs et al., 2017). As opposed to the central nervous system and the heart, abdominal organs and skeletal muscle do not appear to alter metabolic fuel preferences during the administration of sodium‐BHB infusion during basal conditions (Lauritsen et al., 2020) but any direct protein‐sparing capabilities of ketosis have yet to be investigated. We have previously demonstrated protein‐sparing effects during acute inflammation with effects more pronounced on the inflammation augmented protein breakdown (Thomsen et al., 2018). Therefore, we aimed to investigate the direct skeletal muscle protein turnover and metabolism, blood flow, and intramyocellular protein signaling by local infusion of sodium‐BHB. Protein turnover is estimated with inferences measured for both protein synthesis and breakdown rates.

We used single‐leg intervention with the opposing leg acting as a control. We hypothesized that an acute increase in BHB concentration would be associated with protein sparing in healthy males.

## 2 MATERIALS AND METHODS

### 2.1 Design

The study was conducted in a paired design with each subject being investigated on one single occasion with one leg being infused with BHB and the opposing leg acting as a control. Participants were blinded as to which leg was interventional—left or right. Allocation of the interventional side was by simple 1:1 randomization through a closed envelope opened on study day and done sequentially as participants were enrolled. Laboratory technicians, investigators, and other staff were not blinded to interventions side allocation. All investigations were done at the research facilities at the Department of Internal Medicine and Endocrinology, Aarhus University Hospital. The study was approved by The Danish Ethical Committee (1‐10‐72‐87‐15), registered at Clinicaltrials.gov (NCT01461603) and performed in accordance with good clinical practice guidelines and following the principles of the Declaration of Helsinki.

### 2.2 Participants

A total of 10 participants were recruited from notice on a study participant recruitment site. All participants had an inconspicuous medical history and were medically examined including normal screening results. All participants gave oral and written signed consent. Inclusion and exclusion criteria are listed in Supporting Information—in short participants were males of sound health, aged between 21 and 40 years, and had not undertaken participation in studies applying ionizing radiation with the latest year or had skeletal muscle biopsies taken. Participants did not suffer from diabetes, epilepsy, or other known systemic diseases.

### 2.3 Intervention

Infusions of a racemic mixture of 7.5 w/v % sodium beta‐hydroxybutyrate (BHB) (Gold Biotechnology Inc.) at 0.12 g/kg/h and isovolumetric isotonic saline (*Natriumklorid Fresenius Kabi 9 mg/mL*; Fresenius Kabi). The sodium‐BHB solution was adjusted to pH = 7.0 by HCl (Aarhus University Hospital Pharmacy) and double sterile filtered along with contamination tests with Total Aerobic Microbial Count and Total Yeast and Mold Count.

### 2.4 Experimental protocol

**FIGURE 1:**
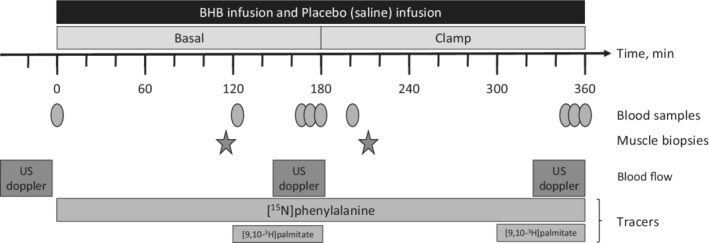
Flowchart of the study day. PHE, phenylalanine; US, ultrasound.

Participants arrived in an overnight fasted state at 6.30 am with prior instructions of abstaining from all physical exercise and sports for 48 h before the study day. Upon arrival baseline, ultrasonic estimation of prior differences in blood flow was performed by ultrasound (Vivid e; GE). Bilateral placement of biluminal catheters (BD Careflow) in aa. Femoralis and vv. Femoralis by Seldinger technique in local anesthesia (Lidocaine [*Xylocain*]; AstraZeneca) with visualization through ultrasound as described elsewhere (Gjedsted et al., 2011). Arterial catheters were placed in the cranial direction and BHB/Placebo were infused through the caudal lumen in the catheter, that is, caudally to the sampling site in the artery. The study day flowchart is depicted in Figure 1.

### 2.5 Protein flux

Through a catheter placed in a cubital vein infusion of [^15^N]‐phenylalanine (10 mg/ml) (Cambridge Isotope Laboratories) at 0.75 mg/kg/hour with priming of the pool by a bolus injection of [^15^N]‐phenylalanine (0.75 mg/kg) was made. Enrichments of ^15^N‐phenylalanine were measured by high‐performance liquid chromatography(HPLC) as their t‐butyldimethylsilyl‐ether derivatives under electron ionization conditions using l‐[^2^H_8_]phenylalanine as an internal standard (Nair et al., 1995). The rate of appearance and rate of disappearance of phenylalanine tracer was calculated as previously described (Copeland & Nair, 1994). These were the primary outcome of this study. All ultrasound blood flow estimates were obtained in triplicates.

### 2.6 Palmitate flux

At 120 and 300 min, an unprimed continuous infusion of albumin‐bound (Gjedsted et al., 2011; Nair et al., 1995) [^3^H]‐palmitate (0.30 μCi/min; GE Healthcare) at a rate of 23 ml/h was infused over 60 min. Blood samples were obtained in triplicates in the final 20 minutes of the basal period (time 0–180 min) and clamp period (180–360 min) with measurements of concentration and specific activity determined by HPLC (Miles et al., 1987) applying [^2^H_31_]‐palmitate as internal standard (Jensen et al., 1988).

### 2.7 Hyperinsulinemic‐euglycemic clamp

Plasma glucose was maintained at approximately 5 mM by adjusting the glucose (*Glukose “SAD” 200 g/L*; SAD Amgros I/S) infusion rate. Plasma glucose was measured at 10‐min intervals (Beckman Instruments). Insulin (*Actrapid*; Novo Nordisk) was infused at 1.0 mU/kg/min.

All blood flow estimates were obtained in three duplicate sets separated by 10 min and recorded in data analysis as their mean.

### 2.8 Muscle biopsies and western blots

Muscle biopsies were obtained simultaneously under local anesthesia (Lidocaine [*Xylocain*]; AstraZeneca) from the lateral vastus quadriceps muscles bilaterally using Bergström biopsy needles. Biopsies were snap‐frozen in liquid nitrogen and stored at −80°C until analysis.

Homogenization of muscle tissue was performed in an ice‐cold buffer at pH = 7.4 containing MgCl_2_ (1 mM), NaCl (137 mM), NaF (20 mM), CaCl_2_ (1 mM), NaVO_4_ (2 mM), Na_4_P_2_O_7_ (10 mM), nicotamide (5 mM), tyramide signal amplification (10 μM), hydroxyethylpiperazineethanesulfonic acid (50 mM), Halt Protease Inhibitor Cocktail (1%), NP‐40 (1%), EDTA (5 mM), and glycerol (10%). By centrifugation at 14,000 *g* for 20 min at 4°C, insoluble materials were removed, and sample adjustment to equal concentration was done with milli‐Q water before proteins were denatured by mixing with Laemmlli's buffer, and heating at 95°C for 5 min. Control for equal loading was performed using stain‐free technology (Gürtler et al., 2013).

Transference of proteins was done by Trans‐Blot® Turbo Transfer System (BioRad) and subsequent visualization by SuperSignal® West Dura (Thermo Scientific) in combination with ChemiDoc MP Imaging System (BioRad) and quantified using BioRad®, Image Lab 4.0.1.

All proteins were tested as the ratio of phosphorylation over total protein content on the same membrane. Selected targets were Akt (also known as protein kinase B), mechanistic target of rapamycin complex 1 (mTORC1), eukaryotic translation initiation factor 4E (eIF4E)‐binding protein 1 (4E‐BP1), and Light Chain 3B (LC3B) –Thr^308^; Akt #9275/panAkt #4691; CST. Ser^2448^ mTOR #2972/mTOR #2971; Cell Signaling Technologies (CST). Nonphosphorylated Thr^46^ 4E‐BP1 #4923/4E‐BP1 #9644; CST. LC3B‐II/I #3868; CST.

### 2.9 Sample size

We have previously found a difference in means of point estimates for net phenylalanine fluxes of ∼10% (Bach et al., 2013). Based on data from that study and specifying *α* = 0.05 and *β* = 0.2 (power 0.8) with a given correlation of 0.5 our calculation with a two‐sided paired Student's *t* test gave a sample size of 10 participants.

### 2.10 Statistical methods

Data were analyzed using repeated measurement restricted maximum likelihood linear mixed effect modeling with intervention (BHB/Placebo) and period (basal/clamp) as factors and their interaction. Inferences were adjusted for a small sample size. Data are presented as means with 95% confidence intervals (CI) or as otherwise specified. If violation of the assumption of normally distributed residuals were found data were transformed as appropriate. As was the case if violation of the homoscedasticity assumption was violated. Heteroscedasticity was taken into account if present after transformation. *p* values of <0.05 were considered statistically significant. Data on phenylalanine dynamics were erroneous in the case of two participants and therefore omitted. All statistical analyses and graphics were performed with Stata (StataCorp. 2019. Stata Statistical Software: Release 16.).

## 3 RESULTS

Ten participants included had a median age of 24 (range 20–33) years and a median BMI of 22.1 (range 19.7–24.6) kg/m^2^.

**FIGURE 2:**
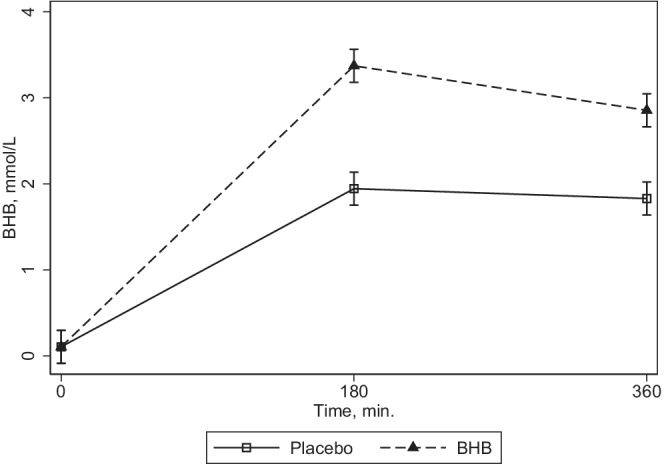
Plasma beta‐hydroxybutyrate concentration. *N* = 10. Plasma BHB concentration over time is shown for the interventions, sodium‐BHB infusion (BHB), and Saline (Placebo). Data were analyzed by repeated measurements multilevel modeling (mixed) with period and interventions as factors. Interaction term *p* > 0.0005. BHB, beta‐hydroxybutyrate.

BHB concentrations increased in both legs, but the increase was substantially larger in the intervention leg (interaction term *p* < 0.001) with a difference of 1.4 (1.0, 1.8) mM, *p* < 0.001 at end of the basal period and 1.0 (0.7, 1.3), *p* < 0.001 mM at the end of the clamp period as depicted in Figure 2. No important harms or adverse events were observed.

### 3.1 Amino acid kinetics

**FIGURE 3:**
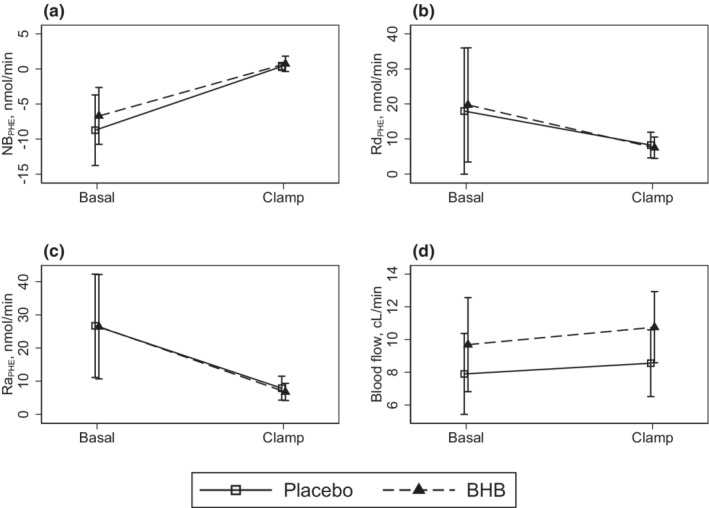
Amino acid tracer phenylalanine turnover and blood flow. *N* = 8. Data are shown as means with 95% CIs. Data were analyzed by repeated measurements multilevel modeling (mixed) with period and interventions as factors. BHB, beta‐hydroxybutyrate; CI, confidence interval; NB, net balance; PHE, phenylalanine; P, plasma; Ra, rate of appearance; Rd, rate of disappearance.

Net phenylalanine balance (NB_PHE_) estimates were numerically ∼30% higher in the BHB intervention leg compared with Placebo leg with effects being −6.7 (−10.8, −2.7) vs −8.7 (−13.8, −3.7) nmol/min, *p* = 0.52 and a slightly differing synthesis rate of 19.7 (3.4, 36.0) vs 18.0 (0.05, 36.0) nmol/min, *p* = 0.91 for BHB vs Placebo. Phenylalanine rates of appearance were 26.4 (10.7, 42.2) vs 26.7 (11.1, 42.3) nmol/min, *p* = 0.79 for BHB and Placebo, respectively, Figure 3a–c. Estimates of fluxes were 2–3‐fold higher in the basal state compared with the insulin‐stimulated clamp period, *p* = 0.02 and *p* < 0.005, respectively, as shown in Table 1 and Figure 3b,c. Blood flow estimates were ∼20% higher with BHB vs Placebo, *p* = 0.03, and equally substantially higher, *p* = 0.007, during insulin infusion in the clamp period as shown in Table 1 and Figure 3d.

**TABLE 1:** Protein dynamics, blood flow, glucose differences, and intramyocellular signals

	BHB		Placebo		Intervention (BHB vs placebo), *p* value	Period (basal vs clamp), *p* value
NB_PHE_, nmol/min						
Basal	−6.7	(−10.8, −2.7)	−8.7	(−13.8, −3.7)	0.52	<0.001
Clamp	0.7	(−0.4, 1.8)	0.4	(−0.2, 0.9)		
Rd_PHE_, nmol/min						
Basal	19.7	(3.4, 36.0)	18.0	(0.0, 36.0)	0.91	0.02
Clamp	7.5	(4.5, 10.5)	8.3	(4.6, 11.3)		
Ra_PHE_, nmol/min						
Basal	26.4	(10.7, 42.2)	26.7	(11.1, 42.3)	0.79	<0.001
Clamp	6.8	(4.2, 9.3)	7.9	(4.3, 11.5)		
Blood flow, ml/min						
Basal	968	(681, 1257)	790	(543, 1038)	0.03	0.07
Clamp	1076	(858, 1293)	856	(652, 1059)		
RaPALM, μmol/min						
Basal	127	(96, 158)	152	(113, 190)	0.17	<0.001
Clamp	32	(22, 41)	40	(26, 55)		
Glucose AV‐diff, mmol/L						
Basal	0.03	(−0.06, 0.13)	0.02	(−0.04, 0.08)	0.63	<0.001
Clamp	0.62	(0.21, 1.04)	0.76	(0.31, 1.21)		
Thr^308^Akt/Akt						
Basal	18.6	(−8.5, 45.7)	25.1	(−21.7, 71.8)	0.62	<0.001
Clamp	167.3	(88.8, 245.9)	115.2	(60.0, 170.4)		
Ser^2448^ mTOR/mTOR						
Basal	0.7	(0.5, 0.8)	0.8	(0.6, 1.0)	0.95	<0.001
Clamp	1.4	(0.9, 1.9)	1.2	(0.8, 1.6)		
Thr^46^ non‐p 4E‐BP1/4E‐BP1^a^						
Basal	0.21	(0.19, 0.24)	0.24	(0.17, 0.32)	0.35	<0.001
Clamp	0.48	(0.31, 0.64)	0.63	(0.27, 0.99)		
LC3 II, 10^−5^						
Basal	588	(516, 660)	570	(498, 642)	0.68	<0.001
Clamp	377	(305, 449)	369	(291, 447)		

*Notes*: *N* = 8. Data are shown as means with 95% CIs. Data were analyzed by repeated measurements multilevel modeling (mixed) with period and interventions as factors including their interaction with corresponding *p* values. All intramyocellular signals are noted in arbitrary units, that is, ratios.

Abbreviations: Akt, protein kinase B; AV, arterio‐venous; BHB, beta‐hydroxybutyrate; CI, confidence interval; mTOR, mammalian target of rapamycin; LC3 II, light chain protein 3; NB, net balance; Non‐p 4E‐BP1, nonphosphorylated eukaryotic translation initiation factor 4E (eIF4E)‐binding protein 1; PALM, palmitate; PHE, phenylalanine; Ra, rate of appearance; Rd, rate of disappearance.

^a^ Reciprocal ratio noted for simplicity since nonphosphorylated antibodies were used.

### 3.2 Palmitate kinetics

Lipolysis, estimated by palmitate rate of appearance, was comparable between the legs with a ∼16% lower rate in the BHB intervention leg vs Placebo leg, 127(96, 158) vs 152(113, 190) μmol/min, *p* = 0.17, see Table 1. Hyperinsulinemia resulted in equally suppressed lipolysis with both interventions, with BHB intervention 127 (96, 158)–32 (22, 41) μmol/min and Placebo 152 (113, 190)–40 (26, 55) μmol/min, *p* < 0.0005.

Similarly, arterio‐venous differences of nonesterified fatty acids were unaffected by intervention and miniscule with BHB vs Placebo in the basal period of −0.005 (−0.01, 0.004) vs −0.007 (−0.01, −0.002), *p* = 0.88. The higher NEFA extraction in skeletal muscle, *p* = 0.001, in the clamp period was unaffected by the intervention (no interaction, data not shown).

### 3.3 Western blots

**FIGURE 4:**
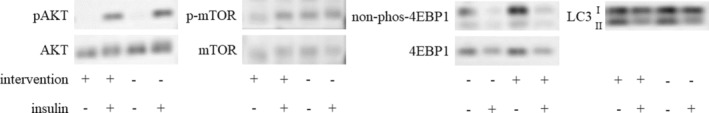
Representative western blots of muscle biopsies. *N* = 10. Results are shown as the proportion of phosphorylated target protein in relationship to the total target protein. Data were analyzed by repeated measurements multilevel modeling (mixed) with period and interventions as factors. 4EBP1, eukaryotic translation factor 4E‐binding protein 1; LC3, light chain 3; mTOR, mammalian target of rapamycin.

Hyperinsulinemia resulted in increased phosphorylation of Akt and mTOR with reduced nonphosphorylation of 4E‐BP1 and LC3B‐II, all *p* < 0.0005, but this effect of insulin was not altered by administration of BHB (see Table 1 and Figure 4).

## 4 DISCUSSION

This study was designed to investigate any direct effects of BHB on protein and palmitate fluxes in human skeletal muscle. Our hypothesis was that direct infusion of sodium‐BHB would decrease the net loss of protein measured by a phenylalanine tracer. We did not find evidence to support nor reject our hypothesis.

### 4.1 Skeletal muscle protein turnover

Net phenylalanine balance estimates were numerically ∼30% higher in the BHB intervention leg compared with the Placebo leg although far from statistically significant. Despite being inconclusive on statistical and maybe also clinical significance, these findings are not necessarily discordant with our previous findings in a systemic model of acute inflammatory disease showing protein‐sparing effect of supraphysiological ketosis (Thomsen et al., 2018). A major difference between the studies may relate to the intensity of the catabolic stimulus imposed. Both systemically and locally induced inflammation increase net phenylalanine release (Bach et al., 2013) and it may be hypothesized that a certain catabolic stimulus is needed for ketosis to alleviate the accompanying increased skeletal muscle breakdown, although there may be stimulus‐specific differences between cytokine and metabolic responses to e.g. TNF‐α and LPS. Whether suppressing muscle protein breakdown is beneficial or not warrants careful consideration in the sense that this is definitely the case in catabolic states, for example, inflammatory disease, but maybe not so in resting states where increased muscle protein synthesis rate is accompanied by increments in the protein breakdown rate (Phillips et al., 1997). Such accompanying increase in protein degradation may be important for the adaptation and impairment of the function of the proteasome is known to impede muscle growth and muscle contractile function (Kitajima et al., 2014).

As expected, hyperinsulinemia per se improved net phenylalanine balance substantially (Meek et al., 1998; Rittig et al., 2016). In alignment with this observation, intramyocellular insulin signaling to protein synthesis was not affected by the difference in BHB concentrations but significantly affected during the hyperinsulinemic‐euglycemic clamp.

### 4.2 Ketosis

Our study has limitations with the most conspicuous one being the substantial systemic spillover of BHB. The magnitude of this phenomenon implies that our study could perhaps be viewed more as a dose–response study comparing two relatively high levels of BHB close to signal saturation, rather than a traditional placebo‐controlled study. While this noticeable risk of being close to saturation of the hydroxycarboxylic acid 2 receptor (Husted et al., 2017), a BHB gradient of 1.4 mM may be clinically meaningful. Furthermore, we did see expected and significantly affected expression of anabolic intramyocellular signaling pathways with insulin stimulation, and blood flow differences between the legs suggesting that meaningful metabolic changes could indeed be investigated. In that context, the present results do not refute the hypothesis that acute ketosis may have direct protein‐sparing and lipolytic effects on skeletal muscle. Future studies need to carefully address the risk of a systemic spillover when investigating direct effects on skeletal muscle when an intraindividual placebo‐controlled design is performed.

### 4.3 Blood flow

We found differences in regional blood flow and the ∼20% higher blood flow rate with BHB compared with Placebo may be meaningful in other clinical settings. This finding is discordant with a study utilizing multiorgan ^15^O–H_2_O perfusion positron emission tomography that did not find augmented blood flow to either abdominal organs or skeletal muscle after systemic intravenous BHB administration (Lauritsen et al., 2020). Another study comparing Na‐BHB and sodium bicarbonate reported no differences in blood flow during basal conditions, whereas skeletal muscle blood flow increased by ∼15% when BHB was combined with hyperinsulinemia (Walker et al., 1991). It remains possible that the high sodium content of the ketone salts may affect hemodynamics (Brian et al., 2018) and infusion of sodium‐BHB‐inducing supraphysiological levels of BHB has been shown to increase blood flow to vital organs, primarily the brain and heart (Gormsen et al., 2017; Svart et al., 2018). It is uncertain whether ketones may alter blood flow through vasodilation or other mechanisms. There is evidence that tissue blood flow and BHB uptake depends on the local metabolic rate in, for example, the central nervous system and exercising vs nonexercising muscle (Mikkelsen et al., 2015); these effects probably involve NO‐induced vasodilation (Avogaro et al., 1999) and prostaglandin I2 release (Avogaro et al., 1996). Reduced blood flow may be involved in the development of sarcopenia in elderly (Zempo et al., 2017) or obese people (Freitas & Katsanos, 2022) and increased muscle blood flow has in the postexercise recovery phase been shown to increase protein synthesis (Biolo et al., 1995). However, blood flow‐restricted resistance exercise has also been shown to augment protein synthesis (Sieljacks et al., 2019). While some stimuli, for example, insulin, may require increased blood flow to exert its net anabolic effect (Fujita et al., 2006), it may be that, for example, exercise is necessary for ameliorative effects of blood flow restriction on protein dynamics (Nyakayiru et al., 2019)—the direction and strength of the association between skeletal muscle blood flow and protein turnover remains to be fully understood. Certainly, the association is subject to modulatory effects of different states like exercise or fed and fasted states. Whether nutritional ketosis is beneficial or not to exercise capacity is yet undetermined (Valenzuela et al., 2020) even though BHB is readily extracted from circulation in both resting and working striated muscle (Cox et al., 2016). The resting muscles and therefore lower energy needs may, at least in part, explain the lack of protein‐sparing effect of BHB as also indicated by the minute glucose extraction and comparable lipolysis rates as evaluated by palmitate flux.

## 5 CONCLUSION

We failed to detect any significant direct metabolic effects of BHB in the leg; this failure in all likelihood relates to a combination of a substantial systemic spillover of BHB to the Placebo leg and the relatively limited number of subjects studied.

## AUTHOR CONTRIBUTIONS

Conceptualization: H.H.T., J.F.O., E.B., J.O.J., and N.M. Data curation: H.H.T., J.F.O., R.A., B.R.R.N., T.S.V., M.V.S., N.R., and E.B. Contributed data and formal analysis: H.H.T., M.J., N.J., E.B., and N.M. Writing—original draft preparation: H.H.T. and N.M. Writing—final draft and review: All. Project administration: H.H.T., J.F.O., E.B., and N.M. Funding acquisition: N.J., J.O.J., and N.M. The final version of the manuscript was read and approved by all authors.

## FUNDING INFORMATION

Grant no. 0603‐00479B rewarded to NM from the Independent Research Fund, Denmark—https://dff.dk/en/front‐page?set_language=en. The funder played no role in study design, data collection, or analysis, decision to publish, or preparation of the manuscript.

## CONFLICT OF INTEREST

The authors declare that they have no conflict of interest.

## Supporting information


**Supporting Information**.
