# Supplementary material for: Investigating effects of sodium beta‐hydroxybutyrate on metabolism in placebo‐controlled, bilaterally infused human leg with focus on skeletal muscle protein dynamics
Source: Physiol Rep. 2022 Aug 19;10(16):e15399. doi: 10.14814/phy2.15399 (PMC9391664; doi:10.14814/phy2.15399)
Supplement: Supplementary file 1 — Supporting Information. [file PHY2-10-e15399-s001.pdf]

Ketosis and protein dynamics – leg study

Article title Investigating effects of sodium beta-hydroxybutyrate on metabolism in placebo-controlled, bilaterally infused human leg with focus on skeletal muscle protein dynamics

Article ID PHY215399

Clinicaltrials.gov NCT01461603

Danish Ethical Committee 1-10-72-87-15

Inclusion criteria

Inclusion criteria were age 21-40 years, male sex, no participation in studies ionizing radiation studies or had muscle biopsies taken within previous year. Inconspicuous medical history, particularly no systemic diseases, particularly no diabetes mellitus, seizures or heart disease, and no history with arterial or venous diseases including venous thromboembolisms or increased risk hereof. All subjects had a normal clinical examination, electrocardiogram, and normal biochemical screening with whole blood count, thyrotropin, liver functions tests, renal functions test, electrolytes, coagulation status. Oral and written consent.

Exclusion criteria

Exclusion criteria were allergy to soy or egg, allergy to catheter materials or similar. Acute or chronic systemic disease, particularly diabetes mellitus, skeletal muscle diseases, renal or liver disease, malignancy, hypertension, or arteriosclerosis. Any active skin disease, e.g. rash or infection, was cause for exclusion. Any ongoing substance abuse, legal or illegal. Medical therapy excluding participations were antiplatelet agents, anticoagulants, immunosuppressive therapy. Consent and participation could be withdrawn at any time during the study.
